# Generalized pustular psoriasis in Brazil: A public claims database study

**DOI:** 10.1016/j.jdin.2021.12.001

**Published:** 2022-01-12

**Authors:** Gleison Vieira Duarte, André Vicente Esteves de Carvalho, Ricardo Romiti, Antonio Gaspar, Thaís Gomes de Melo, Cinara Prata Soares, Anna Rita Aguirre

**Affiliations:** a Instituto Bahiano de Imunoterapia – IBIS, Salvador, Brazil; b Hospital Moinhos de Ventos Porto Alegre, Porto Alegre, Brazil; c Unisinos - Universidade do Vale do Rio dos Sinos, Porto Alegre, Brazil; d Dermatology Division, University of São Paulo, São Paulo, Brazil; e Heads in Health - São Paulo, Brazil; f Boehringer Ingelheim do Brasil - Avenida das Nações Unidas, São Paulo, Brazil

**Keywords:** Brazilian public health system, claims database study, DATASUS, epidemiology, generalized pustular psoriasis, psoriasis hospitalization, psoriasis treatment, pustular psoriasis, GPP, generalized pustular psoriasis, ICD, International Classification of Diseases, PV, psoriasis vulgaris, DATASUS, Departamento de Informática do Sistema Único de Saúde, SIA, Sistema de Informações Ambulatoriais, SIH, Sistema de Informações Hospitalares, SUS, Sistema Único de Saúde

## Abstract

**Background:**

Generalized pustular psoriasis (GPP) is a rare and severe phenotype of psoriasis characterized by sudden outbreak of widespread coalescent sterile pustules associated with a spectrum of systemic symptoms.

**Objective:**

We aimed to describe the epidemiology and treatment of GPP in Brazil from the public health care system perspective.

**Methods:**

This was a retrospective public claims database study, using outpatient and inpatient databases, with information from January 2018 to August 2020, based on records of health resource utilization by patients with GPP. Outpatient treatment regimens and fatal inpatient outcomes were described.

**Results:**

In total, 1458 outpatients of all ages were identified, of whom 53% were women. We estimated the GPP prevalence in Brazil to be between 0.7 and 0.9 per 100,000. Acitretin was the most commonly dispensed drug. Of all the outpatients, 769 outpatients could be tracked in the inpatient database, and 151 had hospital admissions during the study period. Of them, 5.3% had a fatal outcome during hospitalization. A primary skin condition or an infection was the most frequent hospitalization cause.

**Limitation:**

The International Classification of Diseases codes for GPP and psoriasis have not been previously validated in this context.

**Conclusion:**

GPP is a rare disease in Brazil and affects individuals of all ages and both sexes. Hospitalizations and disease-related deaths highlight the need for its prompt diagnosis, close medical follow-up, and effective treatment.

**Capsule Summary**

• This article provides an epidemiologic understanding of generalized pustular psoriasis in Brazil through an analysis of the public health system claims database.
• Although a rare disease, generalized pustular psoriasis is associated with hospital admissions and death, which highlights the need for close medical monitoring and treatment, especially during flares.

## Introduction

Psoriasis is a chronic, multisystem, immune-mediated disorder characterized by inflammatory skin and joint manifestations.^1^ Psoriasis includes different phenotypes, of which psoriasis vulgaris (PV) is the most common. Generalized pustular psoriasis (GPP) is a phenotypically, genetically, and histopathologically distinct entity from PV. GPP has been defined by the European Rare and Severe Psoriasis Expert Network as primary, sterile, macroscopically visible pustules on nonacral skin (except when pustulation is restricted to psoriatic plaques). GPP can occur with or without systemic inflammation, with or without concomitant PV, and can be relapsing (>1 episode) or persistent (>3 months).^2^ Patients with pustular psoriasis have poorer quality of life than those with other forms.^3^ GPP is a severe life-threatening condition, and fatal outcomes attributable to the disease or treatment have been reported in 2%-25% of inpatient series.^4^^,^^5^

The estimated GPP prevalence in France and Japan is 0.0002% and 0.0007%, respectively.^4^^,^^6^ A European survey found that GPP corresponds to 4% of all psoriasis cases.^7^ In Brazil, the estimated prevalence of PV varies from 1.2% to 2.5%, but the prevalence of GPP has not been described.^8^, ^9^, ^10^ Assuming the prevalence of GPP in Brazil to be similar to that in other regions, it could be between 18 and 60 cases per 100,000, a rare disease according to the Ministry of Health.^11^

To understand GPP epidemiology in the Brazilian mixed-race population, we aimed to describe its demographics, prevalence, and health care resource utilization, including treatment and outcomes, through an analysis of information from the Informatics Department of the National Public Health System (DATASUS - Departamento de Informática do Sistema Único de Saúde).

## Materials and methods

This cross-sectional study assessed the epidemiology and pharmacoepidemiology of GPP in the Brazilian public health system (SUS - Sistema Único de Saúde). The Brazilian population in 2020 was 211,755,692.^12^ More than 160 million individuals depended exclusively on SUS for health care.^13^ One of DATASUS' databases is the Outpatient Information System (SIA - Sistema de Informações Ambulatoriais).^14^ In SIA, patients are anonymized by individual numbers. We quantified all patients with at least 1 procedure registered in SIA (medical visit, medication dispensing, or procedure) under the International Classification of Diseases, 10th revision (ICD-10), code L40.1 for GPP from January 2018 to August 2020 and estimated the GPP prevalence among SUS users, in the country and each state, normalized by 100,000. The distribution according to age and sex was described.

Furthermore, we calculated the proportion of GPP among all psoriasis phenotypes (L40.0, PV; L40.1, GPP; L40.2, continuous acrodermatitis; L40.3, palmoplantar pustulosis; L40.4, guttate psoriasis, L40.5, arthropathic psoriasis; L40.8, other forms of psoriasis; and L40.9, nonspecified psoriasis).

For treatment description, we calculated the proportion of patients in SIA with L40.1 who had received treatment with either monotherapy or combination therapy dispensed by the pharmacy, independent of follow-up time.

Comorbidities were assessed in the population with L40.1 by obtaining all concomitant ICD codes recorded between January 2018 and August 2020 for this group; the 20 most frequent comorbidities were described.

SIH (Sistema de Informações Hospitalares - Hospital Information System), DATASUS' inpatient database, was also assessed. To detect individual SIA patients in SIH, a data key composed of the patients' postal code, birth date, and sex was used. Among patients with these 3 pieces of information registered, hospitalized individuals could be identified. We checked for duplicate matched pairs (eg, double death) and cleaned the database. All true linkage pairs were validated, and undecided matched pairs were not included. We obtained the number of patients admitted to hospitals and the number of admissions. The admission rate was calculated as follows: (number of hospital admissions)/(population at risk) × 1000. The most frequent admission diagnoses were described. We also obtained the number of deaths among inpatients and the admission diagnoses of patients who died.

## Results

**Fig 1:**
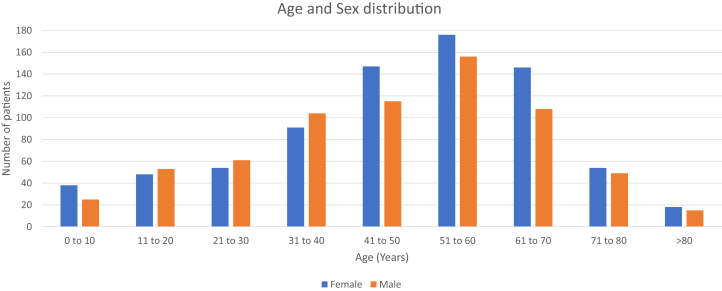
Generalized pustular psoriasis—absolute number of patients according to sex and decade of life.

From January 2018 to August 2020, 30,004 patients had a psoriasis diagnosis in the outpatient database, and of them, 1458 (4.9%) had at least 1 code for GPP, the focus of the present study. The median age was 48 years, with cases of all age groups, and 772 (53%) were women (Fig 1).

The prevalence of GPP in the SUS-dependent population during the study period was 0.89 per 100,000 (0.0009%), and when the whole Brazilian population was considered at risk, it was 0.0007%. The largest proportion of patients was identified in the states of São Paulo (n = 584; 40.1%) and Minas Gerais (n = 204; 14%). The states' prevalence varied from 0% to 0.002% (Table I). The north, northeast, and midwest regions together had 277 cases (19%), whereas the south and southeast regions had 1181 (81%).

**Table I:** Prevalence of GPP in each Brazilian state and federal district

Brazilian states	Inhabitants per federative unit∗	Prevalence (per 100,000 inhabitants)
Acre	894,470	0.4
Alagoas	3,351,543	0.1
Amapá	861,773	0.1
Amazonas	4,207,714	0.4
Bahia	14,930,634	0.4
Ceará	9,187,103	0.2
Espírito Santo	4,064,052	2.2
Federal District	3,055,149	0.1
Goiás	7,113,540	0.5
Maranhão	7,114,598	0.2
Mato Grosso do Sul	2,809,394	0.2
Mato Grosso	3,526,220	0.1
Minas Gerais	21,292,666	1
Pará	8,690,745	0.1
Paraíba	4,039,277	1
Paraná	11,516,840	1.2
Pernambuco	9,616,621	0.2
Piauí	3,281,480	0.1
Rio de Janeiro	17,366,189	0.1
Rio Grande do Norte	3,534,165	0.2
Rio Grande do Sul	11,422,973	1
Rondônia	1,796,460	0.1
Roraima	631,181	0
Santa Catarina	7,252,502	0.6
São Paulo	46,289,333	1.3
Sergipe	2,318,822	1
Tocantins	1,590,248	0.1

∗ From the Instituto Brasileiro de Geografia e Estatistica (IBGE). https://ftp.ibge.gov.br/Estimativas_de_Populacao/Estimativas_2020/POP2020_20210204.pdf. Accessed March 31 2021.

The most frequent concomitant psoriasis diagnosis was PV (L40) in 372 patients (25.5%) (Table II), and the most common concomitant nonpsoriasis diagnoses were mycosis fungoides (n = 76; 5.2%), nonclassified peripheral T-cell lymphoma (n = 69; 4.7%), and chronic viral hepatitis C (n = 69; 4.7%) (Table II).

**Table II:** Comorbidities associated with GPP in an outpatient setting

ICD-10 code	Disease∗	N (%)
L40.0	Psoriasis vulgaris	372 (25.5)
L40.8	Other psoriasis	91 (6.2)
C84.0	Mycosis fungoides	76 (5.2)
C84.5	T-cell lymphoma: other and unspecified T-cell lymphomas	69 (4.7)
B18.2	Chronic viral hepatitis C	69 (4.7)
L40.4	Guttate psoriasis	42 (2.9)
L40.2	Acrodermatitis continua	39 (2.7)
C84.1	Sezary disease	30 (2.1)
L40.3	Pustulosis palmaris et plantaris	27 (1.9)
L86	Keratoderma in disease classified elsewhere	24 (1.6)
L98.9	Disorder of skin and subcutaneous tissue, unspecified	24 (1.6)
L40.5	Arthropatic psoriasis	23 (1.6)
L40.9	Psoriasis, unspecified	14 (1.0)
C80	Malignant neoplasm without specification of site	13 (0.9)
M54.5	Chronic low back pain	12 (0.8)
M25.5	Pain in unspecified joint	10 (0.7)
I10	Essential (primary) hypertension	8 (0.5)
L80	Vitiligo	8 (0.5)
H44.8	Other disorders of globe	8 (0.5)
H40.1	Open-angle glaucoma	7 (0.5)

*ICD*, International Classification of Diseases, 10th revision.

∗ Other comorbidities were associated in <0.5% of the patients.

With regard to outpatients, 1435 patients (98.4%) received monotherapy in enough quantity for at least 1 month of treatment, and acitretin was the most frequently dispensed monotherapy (49.6%), followed by cyclosporine (19%), topical calcipotriol (9.1%), phototherapy (6.9%), methotrexate (5.7%), and topical clobetasol (5%). Although not approved for GPP in Brazil, 4.6% of monotherapies were represented by biologic agents. The most frequently used biologic agent was adalimumab (3.1%).

Combination therapy varied from 2 to 4 concomitant treatments. Most patients (n = 195; 13.4%) received 2 different therapies during a period of at least 1 month. Twenty-nine patients (2.0%) received 3 therapies and 1 patient (0.1%) received 4 therapies simultaneously. Topical calcipotriol was frequently used with acitretin (n = 43; 22.1%), phototherapy (n = 35; 17.9%), clobetasol (n = 33; 16.9%), and methotrexate (n = 18; 9.2%). Phototherapy with acitretin was received by 27 patients (13.8%) and acitretin with clobetasol by 17 patients (8.7%) (Table III).

**Table III:** Treatment frequencies

Monotherapy	N (%)
Nonbiologic systemic therapy	1270 (87.1)
Topical agent	241 (16.5)
Phototherapy	117 (8.0)
Biologic agent	79 (5.4)
Combination of 2 agents	
Topical agent + nonbiologic systemic therapy	96 (6.6)
Topical agent + phototherapy	45 (3.1)
Phototherapy + nonbiologic agent	34 (2.3)
2 topical agents	33 (2.3)
2 nonbiologic systemic therapy	24 (1.6)
Nonbiologic + biologic systemic therapy	8 (0.5)
Topical agent + biologic systemic therapy	1 (0.1)
Combination of 3 agents	
2 topical agents + nonbiologic systemic therapy	16 (1.1)
Topical agent + phototherapy + nonbiologic systemic therapy	12 (0.8)
2 topical agents + phototherapy	2 (0.1)
Phototherapy + 2 nonbiologic systemic therapies	2 (0.1)
Topical agent + 2 nonbiologic systemic therapies	1 (0.1)
Combination of 4 agents	
Nonbiologic + biologic + 2 topical agents	1 (0.1)

**Fig 2:**
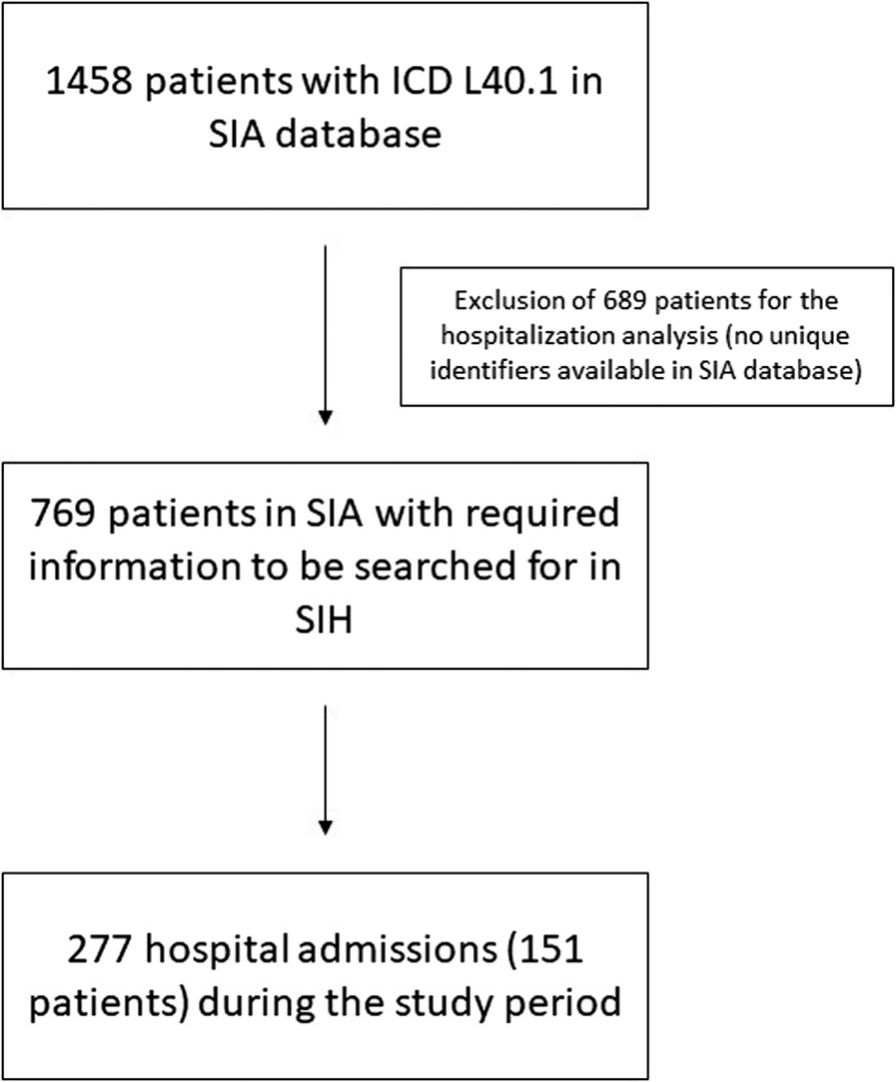
Patient funnel and data linkage from the outpatient database (SIA) to inpatient database (SIH). *ICD*, International Classification of Diseases; *SIA*, Sistema de Informações Ambulatoriais; *SIH*, Sistema de Informações Hospitalares.

Among the 1458 patients in SIA, 769 had the complete data key to be tracked in SIH (52.7%), of whom 151 (19.6%) were hospitalized during the study period, with a total 277 admissions (Fig 2). The admission rate was 360 per 1000. The most frequent admission diagnoses were sepsis or an unspecified bacterial infection (A41.9 or A49.9; n = 14; 5%), atopic dermatitis (L20.9; n = 6; 2.2%), and GPP (L40.1; n = 5; 1.8%).

Eight of the 151 hospitalized patients (5.3%) died during hospital stay, 2.88 per 100 cases. For them, infection was the main cause of admission: sepsis (n = 3; 37.5%) and unspecified bacterial infection (n = 2; 25%). The other causes were malignancy (n = 2; 25%) and liver, gallbladder, or bile duct trauma (n = 1; 12.5%).

## Discussion

This is one of the largest GPP population study and the first assessing GPP in Brazil. Our study identified 1458 outpatients with GPP in DATASUS (4.9% of all psoriasis cases), in line with the European Federation of Psoriasis Patient Associations (EUROPSO).^7^ We calculated the prevalence of GPP considering only SUS dependents (0.0009%). Because Brazilian citizens with private health insurance plans can also use the public system for procedures or medication (eg, acitretin dispensation, frequently not available in the private setting), we also estimated the prevalence to be 0.0007% considering the total Brazilian population to be at risk. In both the scenarios, the prevalence was extremely low, indicating that GPP is a rare disease, and in accordance with previous data.^4^^,^^6^^,^^11^

In this database, all age groups are affected by GPP, as previously described.^15^ The predominance of GPP in adulthood has been demonstrated, and in our sample, the sixth decade of life had the largest number of patients.^4^^,^^16^^,^^17^

Regional disparities (81% of cases in the south and southeast regions and prevalence variation among states) need to be highlighted. Previous prevalence studies in Brazil have identified a higher prevalence of psoriasis in the south and southeast regions than in other regions.^9^ Although genetic characteristics may have an effect, other issues such as variable access to health care or a dermatologist, heterogeneous notification rates, and, possibly, GPP underdiagnosis might be involved.

Most of the concomitant diagnoses were dermatological, either a GPP differential diagnosis or others. This study design did not define whether they are related. This also raises concerns about misdiagnosis because dermatologic diagnoses are not straightforward for general physicians. The precision of ICD-10 code registration might also have interfered. According to the data presented here, overlapping psoriasis phenotypes might also have occurred. Comorbidities usually described in association with psoriasis and GPP, such as metabolic syndrome and its components, were not frequent in our sample.^18^, ^19^, ^20^, ^21^ One explanation is that low-cost medications and primary care visits do not require ICD-10 code recording, making them invisible to the outpatient system.

GPP is a disease without specific treatment guidelines. Recommendations have been adapted from PV treatment protocols.^20^ Our analysis showed that acitretin, cyclosporine, and methotrexate were frequently used as opposed to biologic agents, as described in another publication.^22^ More than 98% of the patients received monotherapy for at least 1 month during the study period. Among them, 59% received acitretin. The Ministry of Health protocol considers acitretin as the first choice for pustular psoriasis, and other options are recommended for moderate-to-severe plaque psoriasis.^20^ Additionally, previous publications have considered cyclosporine and methotrexate as first-line treatments.^21^^,^^22^

Treatment combinations were provided to almost 15% of the patients. Especially for this group, disease control was not easily achieved. This profile, together with a considerable number of deaths, highlights the need for more efficacious GPP therapies. A study of retrospective data from 5 German university centers showed that 31.4% and 27.3% of treated patients reached partial and no response, respectively, reinforcing the need for satisfactory treatment.^17^

Of the total, 53% of outpatients could be tracked in SIH. We found 277 hospital admissions, with no absolute predominance of any cause, but the most frequent cause was bacterial infection, followed by GPP, atopic dermatitis, and other psoriasis forms. As for outpatients, we speculate that GPP diagnosis in the hospitalized patients might also have been inaccurate because it is a rare disease, with low physician awareness. During hospitalizations, the precision of dermatologic diagnoses made by nondermatologists has been reported to be as low as 24%.^23^ If we assume the admission codes of GPP differential diagnosis and its complications as GPP-related admissions, it would be responsible for at least 15% of all the 277 hospitalizations. Of interest, a previous study reported psoriasis as the cause of 4%-37% of all dermatologic admissions.^24^^,^^25^ Our analysis revealed 168 different ICD-10 admission codes, without a clear connection between these and GPP.

Eight patients (5.3% of the 151 hospitalized) died during hospitalization, and almost 3% of the admissions led to deaths, although the relationship between the deaths and GPP could not be established. Historically, in a case series, the deaths of patients with GPP due, directly or indirectly, to the disease and its treatment reached 26 of 106 (32%), and some studies have suggested that fatal outcomes have recently decreased to 2%-7%.^4^^,^^5^^,^^21^ The most frequent cause of admission for these patients was infection, as described in previous articles, which indicates that early and precise identification of GPP and its complications is necessary, especially in patients who require hospitalization.^26^

With regard to limitations, we can mention that this study was based on retrospective data from claims databases, which might have been influenced by misclassification and a selection bias. Although the validity of ICD codes for care-sensitive conditions has been accessed for inpatients in Brazil, 1 major limitation of this study is the absence of specific validation of the GPP and psoriasis ICD codes in the country.^27^ Data quality and precision might have varied among Brazilian federative units because of heterogeneity in reporting procedures, health care facilities, and access to specialized services. The prevalence was estimated using data from a delimited period of time, considering unique patients identified, in 2 possible at-risk population scenarios. Finally, because we did not assess individual patient medical records, a clear relation between the outpatient concomitant ICD-10 codes and hospital admission ICD-10 codes with GPP could not be established.

## Conclusions

This is the first GPP epidemiologic study in Brazil and includes a large portion of its population. It has limitations related to its retrospective database design, but the large sample size reveals important information about GPP in the country. We can conclude that it is a very rare disease that affects individuals of all ages and both sexes, with widespread geographic distribution. Acitretin is largely used as in other countries. The numbers of hospitalizations and deaths were considerable in this cohort, with infections and dermatologic diseases as the main admission causes. Satisfactory disease control in outpatient and inpatient settings is yet to be achieved. Prospective data are expected to better characterize GPP and response to clinical interventions.

## Conflicts of interest

Dr Duarte received grants, consultancy and speaking honoraria from 10.13039/100006483AbbVie, 10.13039/100004326Bayer, Biolab, Boehringer Ingelheim, 10.13039/501100009754Galderma, Janssen, Eli-Lilly, Leo, 10.13039/100011110UCB, 10.13039/100004336Novartis, and 10.13039/100004319Pfizer. Dr Carvalho received grants, consultancy, and speaking honoraria from 10.13039/100006483AbbVie, Janssen, Leo, Boehringer Ingelheim, 10.13039/100004336Novartis, Eli-Lilly, and 10.13039/100011110UCB. Dr Romiti served as a scientific consultant, speaker, or clinical study investigator for AbbVie, Boehringer Ingelheim, Galderma, Janssen-Cilag, Eli-Lilly, Leo-Pharma, Novartis, Pfizer, TEVA, and UCB. Author Gaspar is an employee of Heads in Health. Drs Melo, Soares, and Aguirre are employees of Boehringer Ingelheim do Brasil.

## References

[1] Reich K. (2012). The concept of psoriasis as a systemic inflammation: implications for disease management. J Eur Acad Dermatol Venereol.

[2] Navarini A.A., Burden A.D., Capon F. (2017). European consensus statement on phenotypes of pustular psoriasis. J Eur Acad Dermatol Venereol.

[3] Sampogna F., Tabolli S., Abeni D. (2012). IDI Multipurpose Psoriasis Research on Vital Experiences (IMPROVE) investigators. Living with psoriasis: prevalence of shame, anger, worry, and problems in daily activities and social life. Acta Derm Venereol.

[4] Augey F., Renaudier P., Nicolas J.F. (2006). Generalized pustular psoriasis (Zumbusch): a French epidemiological survey. Eur J Dermatol.

[5] Ryan T.J., Baker H. (1971). The prognosis of generalized pustular psoriasis. Br J Dermatol.

[6] Ohkawara A., Yasuda H., Kobayashi H. (1996). Generalized pustular psoriasis in Japan: two distinct groups formed by differences in symptoms and genetic background. Acta Derm Venereol.

[7] Dubertret L., Mrowietz U., Ranki A. (2006). European patient perspectives on the impact of psoriasis: the EUROPSO patient membership survey. Br J Dermatol.

[8] Duarte G.V., Porto-Silva L., de Oliveira M.F. (2015). Epidemiology and treatment of psoriasis: a Brazilian perspective. Psoriasis (Auckl).

[9] Romiti R., Amone M., Menter A., Miot H.A. (2017). Prevalence of psoriasis in Brazil—a geographical survey. Int J Dermatol.

[10] Ishiy P.S., Silva L.R., Penha M.Á., Handel A.C., Miot H.A. (2014). Skin diseases reported by workers from UNESP campus at Rubião Jr, Botucatu-SP (Brazil). Na Bras Dermatol.

[11] Rare diseases. Brazilian Ministry of Health. https://www.gov.br/saude/pt-br/assuntos/saude-de-a-a-z-1/d/doencas-raras.

[12] Estimativas da população residente no Brasil e unidades da federação com data de referência em 1 de julho de 2020. Accessed March 31, 2021. https://ftp.ibge.gov.br/Estimativas_de_Populacao/Estimativas_2020/estimativa_dou_2020.pdf

[13] Agência Nacional de saúde suplementar. https://www.ans.gov.br/perfil-do-setor/dados-gerais.

[14] DATASUS. https://datasus.saude.gov.br/sobre-o-datasus/.

[15] Twelves S., Mostafa A., Dand N. (2019). Clinical and genetic differences between pustular psoriasis subtypes. J Allergy Clin Immunol.

[16] Jin H., Cho H.H., Kim W.J. (2015). Clinical features and course of generalized pustular psoriasis in Korea. J Dermatol.

[17] Kromer C., Loewe E., Schaarschmidt M.L. (2021). Drug survival in the treatment of generalized pustular psoriasis: a retrospective multicentre study. Dermatol Ther.

[18] Al-Mutairi N., Al-Farag S., Al-Mutairi A., Al-Shiltawy M. (2010). Comorbidities associated with psoriasis: an experience from the Middle East. J Dermatol.

[19] Machado-Pinto J., Diniz M.D., Bavoso N.C. (2016). Psoriasis: new comorbidities. An Bras Dermatol.

[20] Protocolo clínico e diretrizes terapêuticas da psoríase. http://conitec.gov.br/images/Protocolos/Publicacoes_MS/PCDT_Psorase_Final_ISBN_21-08-2020.pdf.

[21] Choon S.E., Lai N.M., Mohammad N.A., Nanu N.M., Tey K.E., Chew S.F. (2014). Clinical profile, morbidity, and outcome of adult-onset generalized pustular psoriasis: analysis of 102 cases seen in a tertiary hospital in Johor, Malaysia. Int J Dermatol.

[22] Hoegler K.M., John A.M., Handler M.Z., Schwartz R.A. (2018). Generalized pustular psoriasis: a review and update on treatment. J Eur Acad Dermatol Venereol.

[23] Davila M., Christenson L.J., Sontheimer R.D. (2010). Epidemiology and outcomes of dermatology in-patient consultations in a Midwestern US university hospital. Dermatol Online J.

[24] Ayyalaraju R.S., Finlay A.Y., Dykes P.J., Trent J.T., Kirsner R.S., Kerdel F.A. (2003). Hospitalization for severe skin disease improves quality of life in the United Kingdom and the United States: a comparative study. J Am Acad Dermatol.

[25] Sen A., Chowdhury S., Poddar I., Bandyopadhyay D. (2016). Inpatient dermatology: characteristics of patients and admissions in a tertiary level hospital in eastern India. Indian J Dermatol.

[26] Kharawala S., Golembesky A.K., Bohn R.L., Esser D. (2020). The clinical, humanistic, and economic burden of palmoplantar pustulosis: a structured review. Expert Rev Clin Immunol.

[27] Cavalcante D.M., Oliveira M.R., Rehem T.C. (2016). Internações por condições sensíveis à atenção primária: estudo de validação do SIH/SUS em hospital do Distrito Federal, Brasil, 2012. Cadernos de Saúde Pública.

